# The influence of KCl concentration on the gelation of myofibrillar protein giant squid (*Dosidicus gigas*) due to molecular conformation change

**DOI:** 10.3389/fnut.2022.1082464

**Published:** 2023-01-04

**Authors:** Fuge Niu, Shuang Ma, Xiuzhen Zhang, Christos Ritzoulis, Yueyue Chen, Weichun Pan

**Affiliations:** ^1^The School of Food Science and Biotechnology, Zhejiang Gongshang University, Hangzhou, China; ^2^Department of Food Science and Technology, International Hellenic University, Thessaloniki, Greece; ^3^China Aquatic Products Zhoushan Marine Fisheries Corporation, Zhoushan, China

**Keywords:** paramyosin, salt concentration, conformation, change, gelation, diffusing wave spectroscopy (DWS)

## Abstract

**Introduction:**

Protein gelation process is of importance in food industry. The objective of this study is to investigate the influence of salt concentration variation, which induced protein conformation change, on protein’s intermolecular interactions and its gelation process.

**Methods:**

Paramyosin has been separated and purified from myofibrillar protein extracted from giant squid. Then Giant squid’s paramyosin molecular mass and intermolecular interactions were quantified by means of light scattering techniques. Finally, the micro-rheology study via diffusing wave spectroscopy (DWS) technique revealed that this conformation change dramatically affected myofibrillar protein gelation process.

**Results:**

The obtained apparent molecular weight (ca 2 × 10^5^ g/mol) suggested that protein molecules existed as dimers, while the second virial coefficient A2 significantly reduced from −3.98456 × 10^−5^ to −5.07575 × 10^−4^ ml mol/g^2^ when KCl concentrated from 0.15 to 1 mol/L. Light scattering data also suggest that paramyosin dimers are stiff, with a persistence length of 120 nm, almost the length of a molecule and independent of salt concentration. Mean-square displacement (MSD) of tracer particles at 5 temperatures with 4 salt concentrations displayed that this conformation change had dramatic effect. Therefore, G’ and G” were remarkably altered with at least one order of magnitude difference owing to this event occurrence.

**Conclusions:**

Paramyosin conformation change due to KCl concentrated enhances attractive interactions with apparent molecular mass increase, which resulted in majority paramyosin molecules (> 99%) in dimeric form and promoted aggregates formation. DWS technique revealed that the conformation change dramatic affected this process characterized by the correlation functions, MSD, and G’ and G”. This study brings forward data on understanding the effect of a major salt supplement, KCl, on the chemical physics of a major muscle protein.

## Introduction

Salt plays a major role in the food industry and is multifunctional. In conventional wisdom, except for the essential role of regulating food flavor to achieve desirable organoleptic characteristics, it is able to modify intermolecular interactions between molecules *via* either the screening effect originating from Coulomb force (1) or binding to the specific positions of bio-macromolecules (2). As a result, protein solubility should strongly depends on ion species and strength. In the protein gelation process, the salt concentration is a common means to modify the morphology of protein aggregates and thereby control the properties of final products (3). In addition, ion has a species effect on protein stability. Hundred years ago, Hofmeister studied the dependency of egg white protein solubility on salt (4), initiating the famous “Hofmeister series,” which later on extended to describe the ion-specific effects on protein stability and other phenomena (5). However, potassium chloride, made up of two Hoffmeister-neutral ions, namely, K^+^ and Cl^–^, should, in principle, not have any influence on protein conformation. However, in 2017, this group reported KCl’s salting-in effect on muscle protein, which has been ascribed to the enhancement of the repulsive intermolecular interactions between protein molecules, with paramyosin changing its conformation from 100% helix to 100% β-sheet when KCl concentration increases from 0.1 M to 0.5 mol/L (6). Understanding in detail the effect of KCl on the paramyosin’s structure is important, as this salt is a prime candidate for the replacement of NaCl in the processing and consumption of muscle food products, given that the consumption of NaCl raises well-known health concerns (7).

In this study, the giant squid was selected due to its abundant and high nutrition (6). However, its drawback is obvious as well, such as bitter taste and high protein solubility with the inferior gelation performance of its mince. Although the effect of salt concentration on this process is reported (8), the effect of composition protein conformation modification has not been available so far, to the best of our knowledge.

In the present study, paramyosin has been separated from the solution of myofibrillar protein extracted from giant squid, and its molecular weight and purity were assessed by sodium dodecyl sulfate-polyacrylamide gel electrophoresis (SDS-PAGE). Since results obtained from the light scattering technique are sensitive to impurities, size exclusion chromatography (SEC) was applied to further analyze the purity of paramyosin. In order to complement the above, the molecular weight of paramyosin and the interactions between the protein individual macromolecules were assessed as well.

Then, diffusing wave spectroscopy (DWS) was applied to track myofibrillar protein gelation with 4 KCl concentrations (0, 0.15, 0.5, and 1 M) under 5 temperatures (25, 30, 35, 40, and 45°C). The salt concentration range covers protein responsibility from no effect to pure screening effect and then to conformation change. The studying temperature was stopped at 45°C because myofibrillar protein molecules denaturation mostly occurred under this value in the heating gelation process (9). This study aims at understanding the mechanism of the KCl effect on paramyosin interactions, thereby probing this protein molecule conformation change under various KCl concentrations. Moreover, the influence of this molecular conformation change on the myofibrillar protein gelation process is uncovered. This study will shed light on meat food process development with the guide of salt-protein interactions in order to achieve desired texture and flavor while gradually substituting NaCl with KCl.

## Materials and methods

### Materials

Glycine, acrylamide solutions with 30% (w/v), SDS, ethylene diamine tetraacetic acid (EDTA), dithio-bis-nitrobenzoic acid (DTNB), 1-anilino-8-naphtha-lene sulfonate (ANS), Triton-X100, N,N,N′,N′-tetramethylethylenediamine (TEMED), and ammonium persulfate (APS) were provided by Macklin Biochemical Co., Ltd. (Shanghai, China). Ready-to-use protein molecular weight standard marker (6.5–200 kDa) was purchased from Sigma (St. Louis, MO). Disodium hydrogen phosphate (Na_2_HPO_4_), potassium dihydrogen phosphate (KH_2_PO_4_), potassium chloride, sodium hydroxide, ethanol, and other reagents were obtained from Hangzhou Jiecheng Biotechnology Co. (Hangzhou, China). All reagents were of analytical purity.

Giant squid, purchased from Zhoushan Second Ocean Fishery Co., Ltd. (Zhoushan, China), were immediately frozen without any treatment after being caught and stored at −20°C for less than 3 months. All squids were deheaded, degutted, and cleaned using water after they were defrosted in the air to room temperature. The mantles with the fins were sealed and stored at −40°C for further experiments.

### Myofibrillar protein preparation

Myofibrillar protein samples were prepared following the procedure described by Hashimoto et al. with minor modifications (10). Ground muscle mixed with buffer A (15.6 mM Na_2_HPO_4_, 3.5 mM KH_2_PO_4_, 10 mM EDTA, pH 7.5) (1:8, w/v) was homogenized using a tissue homogenizer (Homogenizer T25 basic, IKA, Staufen, Germany) at a speed of 12,745 × *g*. Homogenization was performed in an ice bath, as to avoid overheating and consequent protein denaturation; five successive 30 s homogenization cycles were performed, each followed by a 30 s interval. The connective tissues were then removed by filtration using two layers of gauze. The obtained material was centrifuged at 8,000 × *g* for 10 min at 4°C (Biofuge Stratos, Thermo Scientific Inc., Belmont, CA, USA).

The supernatant was removed, and the sediment was resuspended with 2 vol 1% Triton X-100 (w/v). This sample was again centrifuged at 8,000 × *g* for 10 min at 4°C. The residue (the myofibrillar protein precipitate) was washed with 8 vol of buffer A. The mixtures were then centrifuged at 8,000 × *g* for 10 min at 4°C. The supernatant was removed, and the washing procedure was repeated three times. The white paste precipitate was myofibrillar protein.

The white paste precipitate was mixed with buffer B (0.15 M KCl, 15.6 mM Na_2_HPO_4_, 3.5 mM KH_2_PO_4_, pH 7.5) and refrigerated overnight at 4°C. The mixtures were then centrifuged at 8,000 × *g* for 10 min at 4°C. The final pellet, which still carried a lot of aqueous solutions, was collected and stored at −20°C.

### Myofibrillar protein solution preparations

Myofibrillar protein pellets were dissolved in phosphate-buffered saline (PBS) solution and then stayed for 3 h for the protein molecules’ full hydration. This mixture was centrifuged at 10,000 × *g*/10 min/4°C. The supernatants were collected for further study.

### Bradford assay for protein concentration determination

The Bradford assay was utilized in order to determine protein concentration (11). A volume of 1 ml protein solution was mixed with 5 ml Coomassie Brilliant Blue reagent for 5 min, and then, its absorbance values were assessed using a spectrophotometer (UV-2600, Shimadzu, Kyoto, Japan) operating at 595 nm. With the aid of a standard curve obtained from bovine serum albumin, the corresponding concentration values were assessed. All experiments were performed at 25°C, and each sample was assessed at least three times.

### Paramyosin separation and purification

The method to purify paramyosin was described in detail by Zhang et al. (6). In brief, an AKTA purifier (100-fast protein liquid chromatography system) (GE Healthcare, Pittsburg, PA) was used to purify the target protein. The myofibrillar protein sample (6.25 mg/ml) was loaded onto a HiTrap Q FF column (5 ml, GE Healthcare) that had been pre-equilibrated in PBS buffer. The column was washed with 0.1–1 M KCl, using 42 column volumes (CVs) in three consecutive steps, with 0, 0.3, 0.5, and 1 M KCl. The protein concentration was measured based on ultraviolet (UV) absorption at 280 nm. The composition of each fraction (1 ml) was determined using SDS-PAGE. The fractions under peak A (Supplementary Figure 1) were pooled, desalted, and concentrated by centrifugation (10,000 × *g*/10 min/4°C) to 3 mg/ml. Similar operations were carried out for the fractions corresponding to peaks B, C, and D as well. Each sample (500 μl) was then loaded onto a Superdex 200 10/30 GL gel filtration column (24 ml, GE Healthcare) that had been equilibrated with PBS buffer (0.1 M KCl, 15.6 mM Na_2_HPO_4_, 3.5 mM KH_2_PO_4_, pH 7.5) and then eluted with 1.5 CVs of this PBS buffer solution at a rate of 0.5 ml/min. Three fractions were desalted and concentrated. Part of the protein sample was used for assessment, and the rest was dried using a custom-built vacuum freeze-drying system; the obtained powder was packaged and stored at −20°C.

### Paramyosin identification

#### Sodium dodecyl sulfate-polyacrylamide gel electrophoresis

Polyacrylamide gel electrophoresis using dissociating SDS and a Tris–glycine–SDS buffer system in a continuous gel (from 6 to 18%) was performed as described by Zhang using a Mini-Protean™ Tetra system (Bio-Rad Laboratories, Richmond, CA, USA) (6). An aqueous mixture containing 0.1 M Tris–HCl, 4% SDS (w/v), 20% glycerol (v/v), 5 × 10^–5^ mol/L bromophenol blue (3′,3″,5′,5″-tetrabromophenolsulfonephthalein, w/v), and 200 mM dithiothreitol was used to dissociate samples. Then, the resultant samples were heated at 100°C for 5 min and then cooled to room temperature (25°C) before loading. The loading sample concentration was 2 mg/ml. The conditions of electrophoretic runs were 17–25°C for half an hour at 70 V and then for 3 h at 110 V. After electrophoresis, the gel was stained with 0.1% (w/v) Coomassie Brilliant Blue R-250 (Bio Basic Inc., Markham, Canada) in 40% ethanol and 10% acetic acid and then destained with 40% (v/v) ethanol and 10% acetic acid.

#### Purification assessment *via* SEC

A volume of 0.05 g of obtained paramyosin powder was mixed with 10 ml PBS buffer (0.1 M KCl, pH 7.5) at 4°C under stirring for 3 h and then centrifuged at 5,000 × *g* for 10 min at 4°C. The supernatant was collected and diluted down to 1 mg/ml with 0.05 M phosphate buffer and was measured with the Bradford assay. This solution was passed through a 0.45 μm filter (Millex-HA, 25 mm, MCA) to remove dust and was then sonicated in an ultrasound cleaner (KQ-50DE, Shumei Co., Kunshan, Jiangsu, China) at 25°C with an ultrasonic frequency of 40 kHz and an input power of 100 Wm, in order to remove air bubbles. A Shodex SEC/GPC protein KW-804 column (300 × 8.0 mm i.d.), an Agilent 1,260 Quaternary pump with an Agilent 1260 VWD detector (λ = 280 nm) was used to separate and detect the components of the protein solutions. A PBS solution with 0.5 M KCl was used as the mobile phase with a flow rate of 1 ml/min. The elution time was 20 min, and the column temperature was 25°C.

#### Paramyosin solution preparation

Stock solutions of paramyosin were prepared by dissolution of its powder 0.04 g in 40 ml PBS buffer (pH 7.5). Meanwhile, the same PBS buffer was used to prepare 3 M KCl solutions. Solutions with various paramyosin concentrations with 0.15 M KCl were prepared by mixing with the desired volume of the protein solution and the counterpart of the 3 M KCl solution. Similar procedures were also carried out to prepare a series of paramyosin solutions with various concentrations of 0.5 and 1.0 M KCl, respectively.

### Light scattering

#### Dynamic light scattering

The particle size of the purified paramyosin solution was assessed using an ALV/CGS-3 goniometer system (ALV, Langen, Germany). The measurement conditions were 25°C, 90° (the detecting angle), and 1 min (acquiring time for each shooting). Each sample was repeated at least 3 times.

#### Static light scattering

The literature reported that the gyration radius of paramyosin was ca. 38 nm (12), approximately 1/17 of the laser wavelength (632.8 nm). Thus, its static light scattering (SLS) results are independent of the detector angle, (13) and the Debye plot (Eq. 1) could be used to estimate protein molecule mass *M*_*w*_ and the intermolecular interactions characterized by the second virial coefficient *A*_2_ between protein molecules.


(1) $$\frac{K\,C}{R_{\theta }}=\frac{1}{M_{w}}+2\,A_{2}\,C$$

where $R_{\theta }=\frac{I_{\theta }}{I_{0}}$ is the Rayleigh ratio of the scattered to the incident light intensity; *K* is the machine constant defined by $k=N_{A}^{-1}\,{\left(\frac{2\,\pi \,n_{0}}{\lambda^{2}}\right)}^{2}\,{\left(\frac{d\,n}{d\,c}\right)}^{2}$ (2), where *n*_0_ is the refractive index of the solvent at the wavelength of the laser beam, *N*_*A*_ is Avogadro’s number; and $\frac{d\,n}{d\,c}$, the refractive increment, is the derivative of the refractive index *n* with respect to protein concentration *c*.

The refractive index increments were determined using an SEC-3010 DN/DC differential refractometer (WGE Dr. Bures, Dallgow-Doeberitz, Germany), operated at 620 nm and 25°C.

All experiments were carried out at 25°C using salt concentrations of 0.15, 0.5, and 1.0 M. The paramyosin concentrations were varied from 0.01 to 0.1 mg/ml. Thus, the third- and higher-order virial coefficients made no contribution to the measurements. The measurement angle was 90°, and the acquirement time was 1 min.

### DWS analyses

A commercial DWS apparatus (λ = 685 nm, 45 mW, LS Instruments, Fribourg, Switzerland) equipped with a multispeckle echo module for long correlation times was applied in this study (14). A flat glass cell (Hellma 110-OS) with an optical length of 5 mm was used as a container. A Peltier temperature controller (±0.01°C) was used to control the sample temperature. The intensity cross-correlation function was calculated using a digital correlator, based on the intensity assessment *via* a photomultiplier, and the electrical field correlation function *g_1_(τ)* was determined as follows:


(2) g1⁢(τ)=∫0∞p⁢(s)⁢e⁢x⁢p⁢(-(s3⁢l*)⁢k02⁢<△⁢r2⁢(τ)>)⁢ds

where *p(s)* is the probability distribution function of a given path followed by the photon of total length *s*, *s* is the path length, *k*_0_ = *2πn/λ*, with *n* the refractive index of the solvent and λ the wavelength of the laser, *< Δr*^2^*(t) >* is the mean square displacement (MSD) of the scatters, and l* is the photon transport mean free path which depends directly on the sample. The l* of the sample is calculated directly by comparing the count rate (CR) of a reference sample as follows:


(3) $$l=l_{r\,e\,f}\,\frac{C\,R_{s\,a\,m\,p\,l\,e}}{C\,R_{r\,e\,f}}$$

The MSD can be extracted from *g_1_(τ)* inverting Eq. 1. While the storage and loss moduli could be estimated by the Laplace transformation of the MSD (fitted by a polynomial of order 7).

The sample was prepared by blending the desirable amount of myofibrillar protein pellet with PBS buffer containing the desirable KCl concentration. Then, this obtained solution stayed at 4°C overnight for full hydration. Its protein concentration was determined by Bradford assay with the value of 5.6 mg/ml. Approximately 3 ml of solution was moved to the glass cell, which was then mounted on the DWS system. Using the control software, each testing temperature was set and the waiting period was 1–2 min for the system to achieve thermal equilibrium. Then, the DWS measurement was carried out with the transmission mode with an acquirement time of 1 min. Each condition was repeated at least 10 times. The size of the tracer was determined by the dynamic light scattering (DLS) technique.

### Statistical analysis

Values were taken as the mean ± the standard deviation. Descriptive statistics, one-way analysis of variance, and multiple comparisons using Tukey’s test were performed using SPSS software (version 18.0). The significance level was set at *P* < 0.01.

## Results

### Paramyosin separation and purification

Supplementary Figures 1, 3 displayed the myofibrillar protein separations under two consequent columns, HiTrap Q FF 16/10 column (5 ml) and Superdex 200 10/30 GL gel filtration column (24 ml). Protein compositions in the resultant solutions in each marked peak were analyzed by the SDS-PAGE technique and are exhibited in Supplementary Figures 2, 4. The peaks BA, CA, and DA collected by the gel filtration chromatography should be paramyosin with a molecular weight of approximately 97 kDa (6, 15).

The resultant paramyosin was analyzed by SEC to further assess its purity. In Supplementary Figure 5, it can be seen that the majority of protein belongs to paramyosin, since there is only one significant peak while the second small is the system peak. The rough assessment of paramyosin purity due to the peak area shows that its purity should be > 90%. In the separation and purification of paramyosin process, the salting-in effect due to its molecular conformation change results in the significant increase of (*P* < 0.01) *R*_*h*_ of its dimmers. Similar behaviors appear in the mixture of human serum albumin with single-walled and multi-walled carbon nanotubes and the drug molecules interacting with calf thymus DNA (16, 17).

### Intermolecular interactions between paramyosin molecules

**FIGURE 1:**
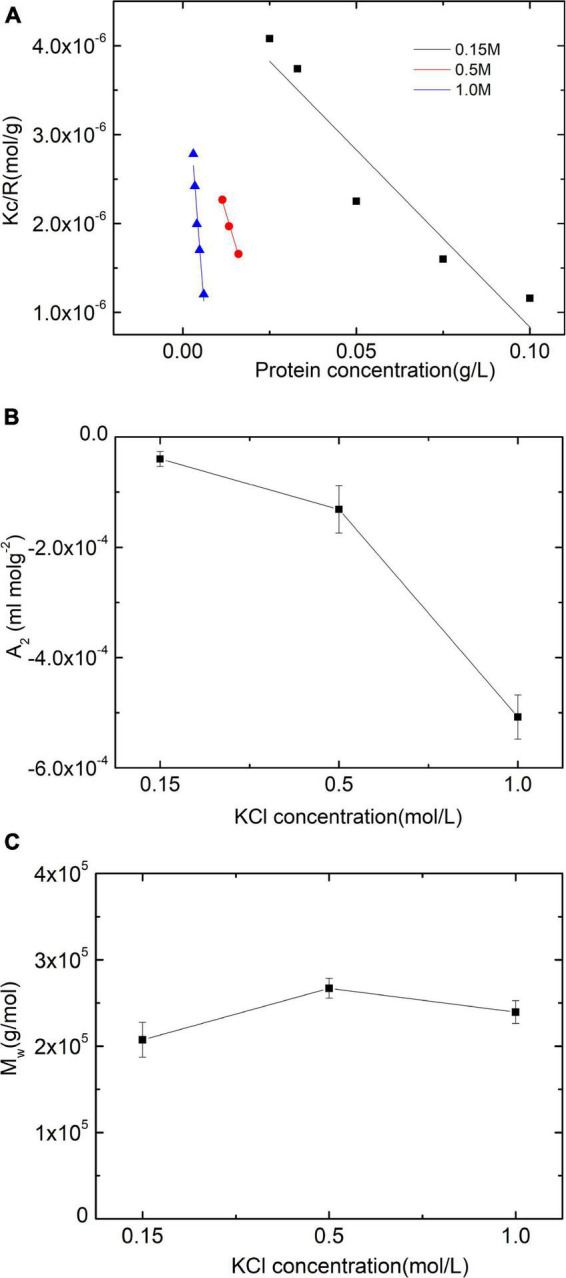
Static light scattering (SLS) assessments of paramyosin. **(A)** Debye plots of paramyosin solutions under three salt concentrations. **(B,C)** Are the corresponding second virial coefficient *A*_2_ and the molecular masses of protein *Mw*.

The intermolecular interactions between paramyosin molecules were assessed by constructing and analyzing a Debye plot (Figure 1A). The slope in a Debye plot is 2*A*_2_ (Eq. 1). The interactions between particles are repulsive when *A*_2_ > 0. While *A*_2_ < 0, these interactions are attractive. The magnitude of *A*_2_ represents the strength of these interactions. If there is not any molecular conformation change with salt concentrated in solutions, the Debye length of the ion carrying the opposite charge around particles becomes short, which will reduce the repulsive double-layer interactions. As a result, the value of the obtained *A*_2_ will decrease.

Thus, from the plot, it can be seen that increasing salt concentrations enhance the attractive interactions between protein molecules (Figure 1B), which is reasonable only if the double-layer force is taken into account (18). The second topic of this study is the apparent *Mw* determination of the isolate as a function of KCl content. Figure 1C shows no particular effect of salt on the apparent *Mw*, at least as far as the effect of self-assembly influences the scattering pattern (0.15 M)-induced apparent *Mw* increase (*P* < 0.01).

**FIGURE 2:**
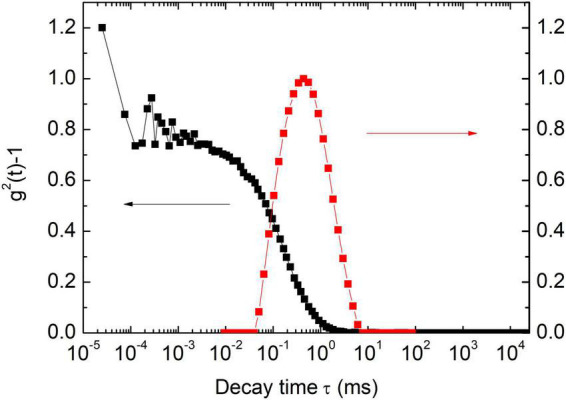
Dynamic light scattering (DLS) autocorrelation function [g^2^(t)-1] for a typical sample (0.15 mol/L KCl) and its corresponding characteristic decay time distribution.

Figure 2 is a typical result of paramyosin DLS assessment, with two populations of particles classified by their hydrodynamic radius (*R*_*h*_). The value of a small particle is ca. 20 nm and is independent of salt concentration. When salt concentration increases from 0.1 to 0.5 M, *R*_*h*_ of large particle population suddenly increases from ca. 95 to 106 nm (*P* < 0.01), a small but reproducible effect.

### The influence of KCl concentration on myofibrillar protein gelation

**FIGURE 3:**
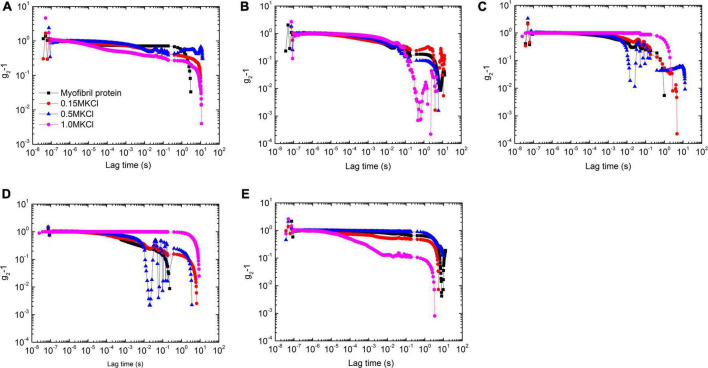
The correlation functions of myofibrillar protein solutions under 5 temperatures [25°C **(A)**, 30°C **(B)**, 35°C **(C)**, 40°C **(D)**, and 45°C **(E)**] with 4 concentrations of KCl [0 mol/L (black square), 0.15 mol/L (red circle), 0.5 mol/L (blue triangle), and 1 mol/L (magenta diamond)].

The introduced KCl with various concentrations dramatically affects myofibrillar protein gelation characterized by the correlation function. At 25°C, a high concentration of KCl facilitated the amplitude decrease of the correlation function, and this function decay was slowly retarded due to KCl concentrated except 0 M (Figure 3A).

When the temperature increased to 30°C, the correlation functions in the short decay time (ca. ≤ 0.1 s) clasped into s master curve for solutions with 4 KCl concentrations (Figure 3B). But they were distinguishable for a long period. Although the characterized decay time was not sensitive to salt concentration except 1 M with the fast decay, the function amplitude had a large value at low concentrations but 0.15 M in which the largest value of amplitude appeared.

When the temperature further increased to 35°C, the correlation function decay became slow down with the large value of amplitude compared with that in 30°C (Figure 3C). The one in 1.0 M solutions had the largest value of amplitude while that in 0.5 M solutions did the smallest one. The corresponding characterized decay time had the same dependency on salt concentration.

When the temperature rose to 40°C, 1 M solutions displayed gel-like behaviors with the value of the correlation function close to 1 throughout almost the whole decay time. The decrease in its amplitude at the end of the decay time measurement came from the echo mode operation (Figure 3D). In addition, 0.5 M solutions showed the retarding as well with the large characterized decay time compared with that at 35°C. All hints that paramyosin conformation change prefers protein binding with slow decay. This conclusion further corroborates by the observations in solutions with the lower salt concentration, in which the fast decay appeared compared with that at 35°C.

But at 45°C, solutions with a low salt concentration (≤0.5 M) decelerated the correlation function while a 1 M solution accelerated it (Figure 3E).

**FIGURE 4:**
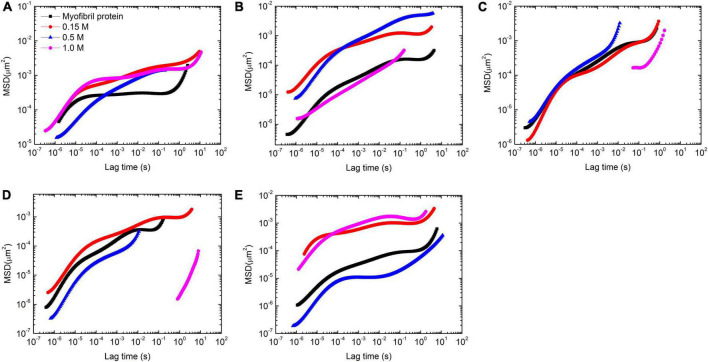
Mean-square displacement (MSD) vs. temperature with 4 concentrations of KCl (0, 0.15, 0.5, and 1 mol/L) at 5 temperatures [25 **(A)**, 30 **(B)**, 35 **(C)**, 40 **(D)**, and 45°C **(E)**] in myofibrillar protein (5.6 mg/ml) gelation process.

In order to study the salt effect in more detail, the corresponding MSD behaviors were calculated (Figure 4). The whole profile of each MSD vs. time curve is usually divided into 2–3 parts discerned by their slope, which indicates that the particles experienced various environments in the whole measurement time. In addition, the plateau and the inflection point could be used to estimate the cage size by the corresponding value in *y*-coordinate with *y* = 6δ^2^, where δ is the value of apparent cage size (19, 20).

Solutions at 25°C showed that high salt concentration had less effect on the short time motion of tracer particles but decelerated their long time motion (Figure 4A). Additionally, the cage size increased from 7 to 11 nm with salt concentrated from 0 to 1 M (Supplementary Table 1).

With the temperature rising to 30°C, the tracer particle motion slowed down in 0, 0.15, and 1 M solutions compared with that at 25°C (Figure 4B). But the opposite trend appeared in 0.5 M solutions. In addition, the speed of this motion had two classes. One includes 0 and 1 M and the rest concentrations of salt are set in the second class with a relatively fast motion compared with the first class. Additionally, the fast motion was associated with a large apparent cage size (Supplementary Table 1).

Further increasing to 35°C, the motion of tracer particles sped up compared with that at 30°C in 4 concentrations of salt. The fast motion speed of tracer particles was 0.5 M > 0 M > 0.15 M∼1 M (Figure 4C). In addition, the apparent cage size was 3 nm except in 1 M with 5 nm. This observation is of interest. The influence of the paramyosin conformation change on the myofibrillar protein gelation process depends on salt concentration.

At 40°C, the motion speed of tracer particles was 0.15 M > 0 M > 0.5 M > 1 M (Figure 4D). In addition, the difference between 1 M and the rest was dramatic in terms of MSD vs. time curve. The apparent cage size followed the same order *via* decreasing from 5 to 2 nm.

The remarkable change occurred at 45°C. The tracer particles moved the fastest in 1 M solutions and then, slightly slowed down in 0.15 M solutions. In 0.5 M solutions, the tracer particles moved the slowest (Figure 4E). Additionally, the fast motion may partially come from the large apparent cage size. Along this salt concentration order, this value decreased from 9 to 7 nm, then to 2 nm, and finally to 1 nm.

The rheological properties of myofibrillar protein changed with salt concentration under these 5 temperatures in terms of G′ and G″ as well.

At 25°C, 0 M solutions were solid-like with G′ large than G″ (Supplementary Figure 6A), and ω_*c*_ (the value of ω for G′ and G″ crossed over) was 1.5 × 10^5^ rad/s. A similar pattern occurred in 0.15 M solutions but G′ decreased while G″ slightly increased compared with that in 0 M. The paramyosin conformation change due to salt concentrated to 0.5 M induced the solution presence solid-like at low ω. The corresponding ω_*c*_ was ca. 7 × 10^3^ rad/s. It is of interest that salt concentrated to 1 M induced both G′ and G″ decrease and ω_*c*_ increase to 4 × 10^4^ rad/s.

At 30°C, G′ and G″ increased in 0 M solutions compared with at 25°C with G′ dominating at low ω (Supplementary Figure 6B). ω_*c*_ was ca. 4 × 10^4^ rad/s. The temperature had less effect on G′ and G″ in 0.15 M, in which the pattern of the relative position between G′ and G″ was the same as that in 0 M and with the same value of ω_*c*_. The paramyosin conformation change did not favor G′ and G″ in 0.5 M solutions. Although the low ω preferred solid-like behavior with G′ dominance over G″, ω_*c*_ reduced to ca. 10^4^ rad/s. But in 1 M solutions, G′ and G″ increased compared with 25°C and were close to each other except at very low ω (ca. 7 rad/s) in which G′ was larger than G″.

At 35°C, in 0 M solutions, G′ was larger than G″ at low ω with ω_*c*_ of ca. 4 × 10^4^ rad/s (Supplementary Figure 6C). The salt concentrated at 0.15 M induced both G′ and G″ increase but their relative position was the same as that in 0 M with ω_*c*_ of ca. 4 × 10^4^ rad/s. The further concentrated salt to 0.5 M with myofibrillar protein conformation change reduced both G′ and G″. Additionally, G′ dominated over G″ at low ω with ω_*c*_ of ca. 3 × 10^4^ rad/s. It is noted that 1 M solutions cannot produce enough data for this analysis.

At 40°C, the salt concentration decreased G′ and G″ at first and then increased again (Supplementary Figure 6D). It is noted that 0 M solutions had ω_*c*_ with the value of ca. 7 × 10^4^ rad/s. Additionally, G′ was larger than G″ when ω less than ω_*c*_. The same pattern was retained in 0.15 M solutions with ω_*c*_ of ca. 3 × 10^4^ rad/s. When salt concentrated to 0.5 M, there were two ω_*c*_s with the values of 300 rad/s and 1.9 × 10^4^ rad/s, respectively. In the middle part of this frequency region, G′ dominated over G″.

At 45°C, 1 M solutions had the smallest value of G′ and G″ (Supplementary Figure 6E), while in 0.5 M solutions, both values were almost the largest. It is noted that 0 M solutions presented the second largest values of G′ and G″. G′ dominated over G″ in all salt concentrations but 0 M, in which ω_*c*_ was 7 × 10^4^ rad/s.

In 0 M solutions, with a temperature rising from 25 to 45°C, G′ and G″ increased, then decreased, and increased again (Supplementary Figure 7A). At 45°C, G′ and G″ attained the maximum values. In addition, the difference between G′ and G″ generally narrowed down with the temperature rising. Additionally, the value of *ω_*c*_* decreased until the temperature reached 40°C and then increased.

In 0.15 M solutions, the patterns of G′ and G″ dependencies on temperature were similar to that in 0 M solutions (Supplementary Figure 7B). However, at high temperatures (≥40°C), G′ dominated over G″ throughout the whole available ω spectra, and the properties of solid-like became more obvious with the temperature increasing.

At 0.5 M conditions with paramyosin conformation change, solutions displayed distinguishable behaviors from the solutions with less salt concentration in terms of G′ and G″ dependencies on ω. At low temperatures (≤35°C), the difference between G′ and G″ was small compared with that in solutions with low salt concentration (Supplementary Figure 7C). Additionally, the temperature had less effect on G′ and G″ in this temperature region as well. With the temperature further increasing, both values rose. But at 40°C, G′ dominated over G″ at low and high ω regions. In contrast, G′ almost dominated over G″ throughout the whole available ω spectra.

In 1 M solutions, G′ and G″ first increased and then decreased with the temperature rising (Supplementary Figure 7D). The difference between G′ and G″ followed the same pattern.

## Discussion

### Cluster existence

The concentrated salt shortens the Debye length to increase the screening effect; thereby the double-layer force decreases. However, this trend contradicts the observation that increases in salt concentration enhance the repulsive interactions with a salting-in phenomenon appearance (6). This last source reports that paramyosin molecule conformation changes from 100% α-helices to 100% β-sheet as KCl concentration increases from 0.1 to 0.5 M, while the value of *A*_2_ increases with KCl, suggesting that the tendency for aggregation is reduced with increasing salt content. This dilemma could be compromised by the fact that > 90% of protein molecules exist in myofibrillar protein solutions by the means of nanoprotein particles (NPPs) (21). Thus, salt concentration affects paramyosin conformation to induce NPP structure modification and thereby influences interactions between NPP. Currently, the strategy to study each component separately to probe the entire food’s properties is still popular; however, this bottom-up pathway should be verified carefully in food research due to implications relevant to the above observation.

The results of the *Mw* determination suggest the following two issues. (1) The apparent *Mw* is approximately twofold of 100 kDa, which hints that the preferred paramyosin quaternary structure is a dimer. This result is consistent with reports in which the apparent *Mw* ranges from 200 kDa to even higher values (22). Since the paramyosin assembly is regulated by intermolecular ionic interactions (23), the presence of SDS, an organic sodium salt that is also a surfactant and a protein denaturant, may induce dimer disassembly into monomers, as verified by the *Mw* of 97 kDa shown by SDS-PAGE. (2) Paramyosin conformation changes induce it to further aggregate, with apparent *Mw* increases. However, the problem with this observation is that all reported values are not multiplies of the *Mw* assessed by SDS-PAGE analyses, with the exception of that in 0.15 M KCl. This observation hints that some paramyosin molecules participate in the self-assembly, which has been reported in the presence of divalent cations (22), even at low concentrations of protein and salt with pH between 6 and 7 (24). This is partly because the apparent *Mw* is calculated using the following formula:


(4) <M>apw=∑k=1nfkMwk

where the superscript *ap* stands for “apparent”; *k* does for the species *k*, and *f* denotes weight fraction (25).

The values of *R*_*h*_ are reasonable. It is because the radius of gyration (*R*_*g*_) of dimer paramyosin could be estimated by $R_{g}^{2}\approx \frac{L^{2}}{12}$ (26) with the value of ca. 37 nm, a good agreement with 41 nm (12). Due to the Kirkwood–Riseman theory, $R_{h}\approx \frac{2}{3}\,R_{g}$ (27), *R*_*h*_ of paramyosin dimer is ca. 25 nm, a good agreement with the present result. The *R*_*h*_ of paramyosin dimer does not depend on the salt concentration (Table 1). It is of interest that protein conformation change results in insignificant alternations of *R*_*h*_ for monomer proteins (*P* ≥ 0.01) but has a serious effect on the *R*_*h*_ of dimers (*P* < 0.01). Indeed, NaCl can play a similar role to induce paramyosin conformation change. Additionally, these behaviors are not new and have been observed in the studies of human serum albumin interacting with drug molecules and other proteins (28, 29).

**TABLE 1:** Summary of dynamic light scattering (DLS) assessments of paramyosin under three salt concentrations.

C_KCl_ (mol/L)	0.15	0.5	1
R_*h*_^1^ (nm)	19 ± 1	23 ± 2	20 ± 3
I^1^ (%)	4.5 ± 0.6*	4.9 ± 0.6**	2.5 ± 0.6***
R_*h*_^2^ (nm)	95 ± 2*	106 ± 3**	105 ± 1**
I^2^ (%)	94.4 ± 1.9	94.9 ± 0.5	96.6 ± 0.6

Within rows, means with different superscripts are significantly different (*P* < 0.01).

Another interesting aspect of this observation is that the dimeric paramyosin molecule is stiff with a persistence length of 120 nm, based on the estimations of *Rg* and *Rh* (27). In addition, the images obtained by electron microscopy corroborate this conclusion with paramyosin molecules being almost straight rods (23).

The dependency of *R*_*h*_ of large particle population on salt concentration suggests that molecular conformation changes, coupled with strong attractive interactions, promote large aggregate formation in paramyosin solutions. Here, the solutions are in a dilute regime since the average distance between dimer paramyosin molecules is ca. 150 nm (0.1 mg/ml). Therefore, the observed large particles due to the entanglement among dimer paramyosin may confidently be ruled out.

### Verification of SLS results

Since the presence of paramyosin aggregate contributes to light scattering results, it is necessary to estimate its magnitude.

For a polarized laser (13), the Rayleigh ratio is $R_{\theta }=\frac{I_{\theta }}{I_{0}}=\frac{\pi^{4}\,\left(n^{2}-n_{0}^{2}\right)\,s\,i\,n^{2}\,\theta }{N\,r^{2}\,\lambda^{4}}$, where *r* is the distance between the sample and detector, and *N* is particle number concentration. The fluctuation theory of light scattering (30) leads to the following:


(5) $$\frac{K\,C}{R_{\theta }}=\frac{1}{P\,\left(\theta \right)}\,\left(\frac{1}{M_{w}}+2\,A_{2}\,C\,P\,\left(\theta \right)+3\,A_{3}\,C^{2}\,P\,\left(\theta \right)+4\,A_{4}\,C^{3}\,P\,\left(\theta \right)+\cdots \right)$$

where *P(θ)* is the shape factor; *A*_3_ and *A*_4_ are the third and fourth virial coefficients. If there are two types of particles with different *R*_*h*_, with the aid of Eq. 5, the ratio of these two types of particles in terms of their number is as follows:


(6) $$\frac{N_{2}}{N_{t\,o\,t\,a\,l}}=\frac{\frac{I_{2}\,\left(\theta \right)}{K_{2}\,M_{w}^{2}\,P_{2}\,\left(\theta \right)}}{\frac{I_{1}\,\left(\theta \right)}{K_{1}\,M_{w}^{2}\,P_{1}\,\left(\theta \right)}+\frac{I_{2}\,\left(\theta \right)}{K_{2}\,M_{w}^{2}\,P_{2}\,\left(\theta \right)}}$$

where subscribes 1 and 2 denote dimer paramyosin and its aggregates, respectively. Furthermore, 1 + 2*A*_2_*CM*_*w*_*P*(θ) + 3*A*_3_*C*^2^*M*_*w*_*P*(θ) + 4*A*_4_*C*^3^*M*_*w*_*P*(θ) + ⋯≈1since the concentrations of paramyosin and its aggregates are low. In addition, we assumed ∂⁡*n*/∂⁡*C*_1_≈∂⁡*n*/∂⁡*C*_2_. If we roughly estimate the *Mw* of paramyosin aggregates by $\frac{M_{w\,2}}{M_{w\,1}}={\left(\frac{R_{2}}{R_{1}}\right)}^{3}$, and with the aid of the definition of *P*(θ) of a spherical particle, Eq. 6 could be rewritten as


(7) $$\frac{N_{2}}{N_{t\,o\,t\,a\,l}}=\frac{\frac{I_{2}\,\left(\theta \right)}{{\left(s\,i\,n\,\left(q\,R_{2}\right)-q\,R_{2}\,c\,o\,s\,\left(q\,R_{2}\right)\right)}^{2}}}{\frac{I_{1}\,\left(\theta \right)}{{\left(s\,i\,n\,\left(q\,R_{1}\right)-q\,R_{1}\,c\,o\,s\,\left(q\,R_{1}\right)\right)}^{2}}+\frac{I_{2}\,\left(\theta \right)}{{\left(s\,i\,n\,\left(q\,R_{2}\right)-q\,R_{2}\,c\,o\,s\,\left(q\,R_{2}\right)\right)}^{2}}}$$

In Eq. 7, the value of *R* is used by *R*_*h*_ and the value of *I* is displayed in Table 1 as the intensity percentage. Therefore, the ratio of paramyosin aggregates in terms of their number concentration could be assessed. The calculation suggests that only ca. 1% (number percentage) of the solute particles are paramyosin aggregates.

In addition, with the aid of Eq. 4, *f*^2^ and *M*_*w*_^2^ (the apparent molecular mass of aggregates) could be roughly estimated as follows:


(8) $$f_{i}=\frac{N_{i}*M_{w}^{i}}{N_{1}*M_{w}^{1}+N_{2}*M_{w}^{2}}$$

The results elucidate that the mass fraction of aggregates is ca. 1–2%, and the apparent molecular mass is ca. 1,000 kDa, which suggests that there are ca. 5 dimer paramyosin particles inside an aggregate.

The obtained *A*_2_ by SLS is an apparent value, which is determined as follows:


(9) A2a⁢p=12⁢(Mwa⁢p)2⁢∑j.k=12fi⁢fj⁢MWj⁢MWk⁢A2j⁢k

where $A_{2}^{j\,k}$stands for the interactions between species *j* and *k*. Due to the low concentration of paramyosin aggregates, the contribution between paramyosin and its aggregates plus that between aggregates could be ignored. Here, we should stress that the *Mw* of paramyosin aggregates may be dramatically overestimated by the proposed method because the geometrical structure of both types of particles is not spherical. Therefore, the assumption of $\frac{M_{w\,2}}{M_{w\,1}}={\left(\frac{R_{2}}{R_{1}}\right)}^{3}$ is not accurate. In addition, the aspect of the molecular weight of aggregate (ca. 1,000 kDa) suggests that this structure is not compact. All induce the values of the number of percentage of aggregates almost equal to that of mass percentage.

This investigation suggests that the intermolecular interactions assessed by SLS mainly come from the contribution of dimeric paramyosin.

### The influences of paramyosin molecular conformation change on rheological properties of myofibrillar protein

Diffusing wave spectroscopy results highlighted the importance of paramyosin conformation change. Zhang et al. revealed KCl concentrated in myofibrillar solution, enhancing the repulsive interaction between protein particles (6), which should speed up the corresponding particle movement with the fast decay of their correlation function (31), contradicting the experimental result. However, the paramyosin conformation alteration provides a solution. The concentrated KCl promoted paramyosin molecule aggregation to induce the particle size increase and thereby slowing down the particle motion.

It is known that salt concentration affects myofibrillar protein composition and its conformation (32). The fact of dramatic increase of β-sheet content in myofibrillar protein when salt concentration jumps from 0.1 to 0.5 M but no further discerned change when salt increases to 0.9 M suggest that this content increase is mainly due to paramyosin conformation change at pH near neutral. Additionally, salt concentration affects the protein denature process at 35°C (33, 34). The paramyosin molecule conformation change induced myofibrillar protein aggregators’ disassembly to speed up their correlation function decay. However, further concentrated salt resulted in attractive interactions increasing to stop this disassembly but instead to induce protein molecules binding, which was verified by the slow decay in 1.0 M solutions. In addition, at 45°C, the gel was destroyed, which hints that the resultant gel gets mature below this temperature. Moreover, paramyosin conformation change induced protein particle aggregation to swell the apparent cage size in solutions. We ascribed this to the attractive interactions increasing due to the salt screening effect, which somehow induces the cage size swelling.

## Conclusion

Paramyosin has been separated and purified from the solution of myofibrillar protein extracted from the giant squid. Then, SLS is applied to paramyosin solutions with three concentrations of KCl. This change enhances attractive interactions with apparent molecular mass increase. DLS results uncover the following two issues: (1) majority paramyosin molecules (>99%) are in dimeric form; (2) paramyosin aggregates are formed, which could be used to further explain some contradictions in the existing literature. Paramyosin molecular conformation change promotes aggregate growth. In addition, the influence of this protein conformation change on the myofibrillar protein gelation process was investigated *via* the DWS technique. Results revealed that the conformation change dramatically affected this process characterized by the correlation functions, MSD, and G′ and G″.

This study brings forward data on understanding the effect of a major salt supplement, KCl, on the chemical physics of a major muscle protein. Paramyosin is versatile because its application ranges from food to medicine and pharmaceutical practices. However, its physicochemical properties are still not fully understood. Our study could fill this gap and shed light on the further study on this protein.

## Data availability statement

The original contributions presented in this study are included in the article/Supplementary material, further inquiries can be directed to the corresponding author/s.

## Author contributions

FN: methodology and investigation. SM: investigation and writing. XZ and YC: investigation. CR: writing—review and editing. WP: conceptualization, supervision, and writing. All authors contributed to the article and approved the submitted version.
