# Supplementary material for: The influence of KCl concentration on the gelation of myofibrillar protein giant squid (Dosidicus gigas) due to molecular conformation change
Source: Front Nutr. 2023 Jan 4;9:1082464. doi: 10.3389/fnut.2022.1082464 (PMC9846250; doi:10.3389/fnut.2022.1082464)
Supplement: Supplementary file 1 [file Data_Sheet_1.docx]

**Supplemental materials**

**The Influences of salt concentration on** **intermolecular interactions between Paramyosin molecules**

Fuge Niu^a^, Shuang Ma^a^, Xiuzheng Zhang^a^, Christos Ritzoulis^a,b^, Yunyun Chen^c^, Weichun Pan^a,*^

^a^ *The School of Food Science and Biotechnology, Zhejiang Gongshang University, Hangzhou, China, 310018*

^b^ *Department of Food Science and Technology,* *International Hellenic University, 57400 Thessaloniki, Greece*

^c^ *China Aquatic Products Zhoushan Marine Fisheries Corporation, No.1,Xinghai Road, Shenjiamen Street, Putou District, Zhoushan, China, 316101*

Figure. S1 Chromatogram of Myofibrillar protein in HiTrap Q FF 16/10 column (5 mL) equilibrated and eluted with PBS buffer (pH 7.5). Column was washed with 0.1–1 mol/L KCl for 42 CVs in three consecutive steps, with 0.3, 0.5, and 1 mol/L KCl, at a flow rate of 3 mL/min and fraction volume 1 mL. Dotted line represents UV absorbance and solid line represents salt concentration profile.


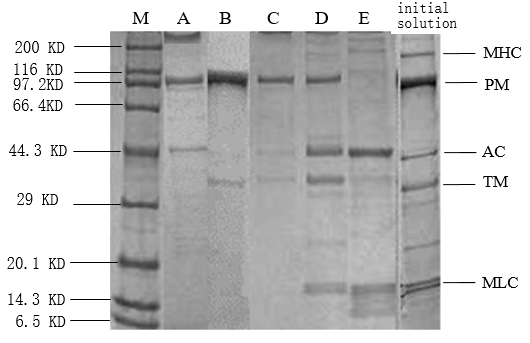


Figure. S2 SDS-PAGE analysis of products due to HiTrap Q FF 16/10 separation. The letters at the top of the figure designate for the various fractions collected in Figure S1 distinguished by the same letters, except for M, which stands for the markers. The last column is for the raw material, which is treated by HiTrap Q FF 16/10 separation.

Figure. S3 Chromatograms of fractions A (A), B (B), C (C), D (D), and E (E) in Superdex 200 10/30 GL gel filtration column (24 mL).


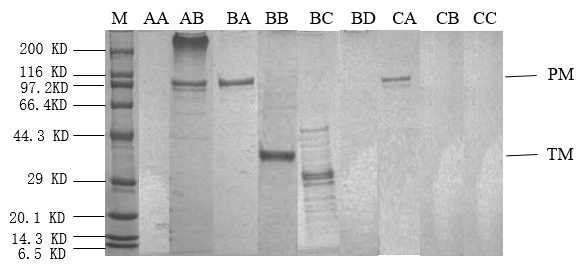


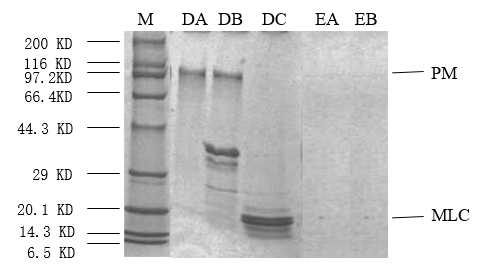


Figure. S4 SDS-PAGE analysis of products due to Superdex 200 10/30 GL gel filtration column separation. The letters at the top of the figure designate for the various fractions collected in Figure S3 distinguished by the same letters, except for M, which stands for the markers.

Figure. S5 Chromatogram of paramyosin in a Shodex SEC/GPC Protein KW-804 column (300 × 8.0 mm i.d.) equilibrated and eluted by PBS buffer (pH 7.5) containing 0.5 mol/L KCl with a flow rate of 1 mL/min with the aid of an Agilent 1260 Quaternary pump with an Agilent 1260 VWD detector (λ = 280 nm).

Table S1 The cage sizes of gel in various heating temperatures with the unit of nm.

| C_salt_ (mol/L) | 0 | 0.15 | 0.5 | 1.0 |
| --- | --- | --- | --- | --- |
| T (^o^C) |  |  |  |  |
| 25 | 7 | 9 | 9 | 11 |
| 30 | 2 | 7 | 7 |  |
| 35 | 3 | 3 | 3 | 5 |
| 40 | 2 | 4 | 2 |  |
| 45 | 2 | 7 | 1 | 9 |

















Figure. S6 The elastic modulus (denoted by solid symbol) and viscous ones (done by empty symbol) with 4 concentrations of KCl (0, 0.15, 0.5, and 1 mol/L) at 5 temperatures (25 (a), 30(b), 35(c), 40(d), and 45^o^C(e)) in myofibrillar protein (5.6 mg/mL)gelation process.


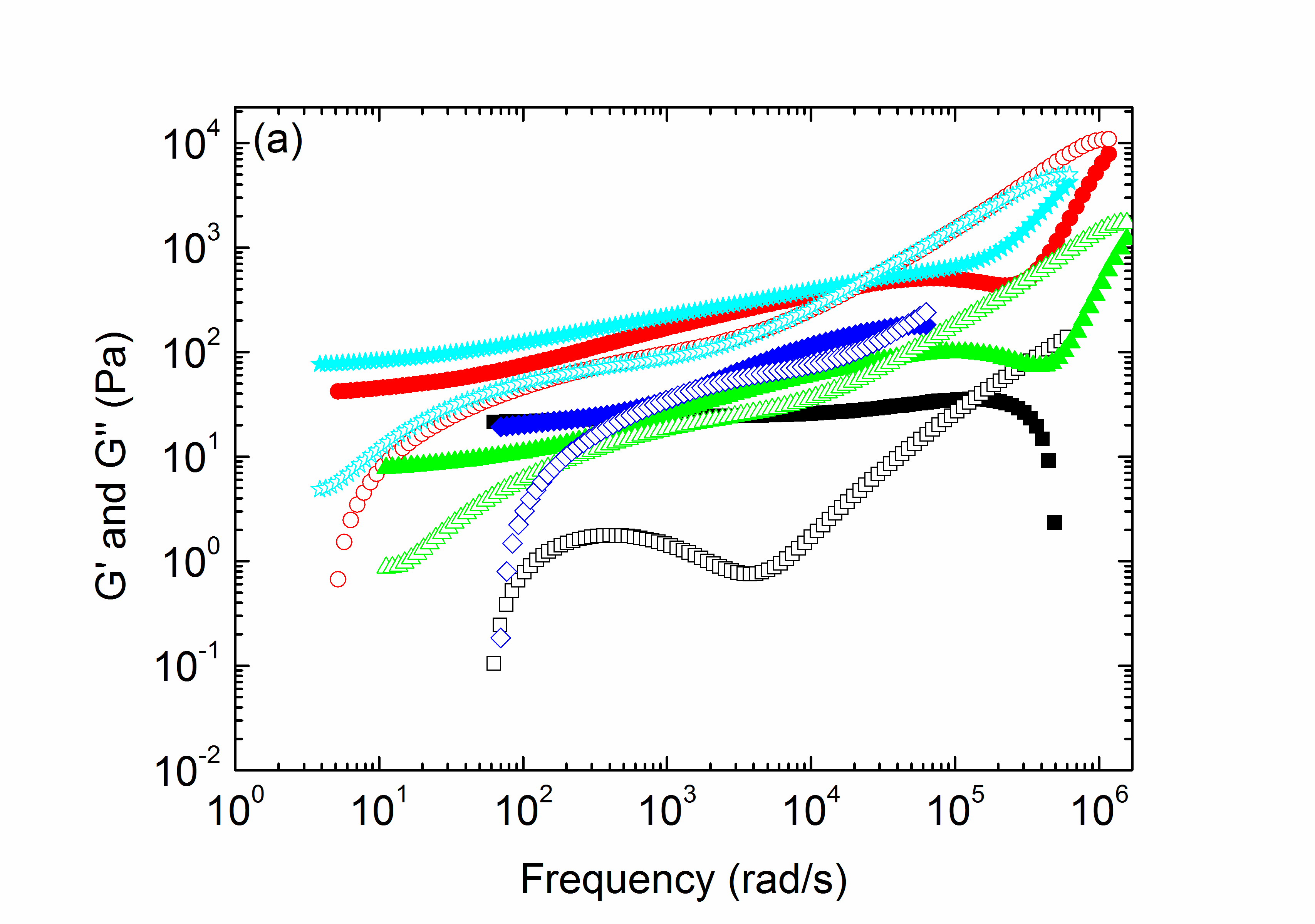


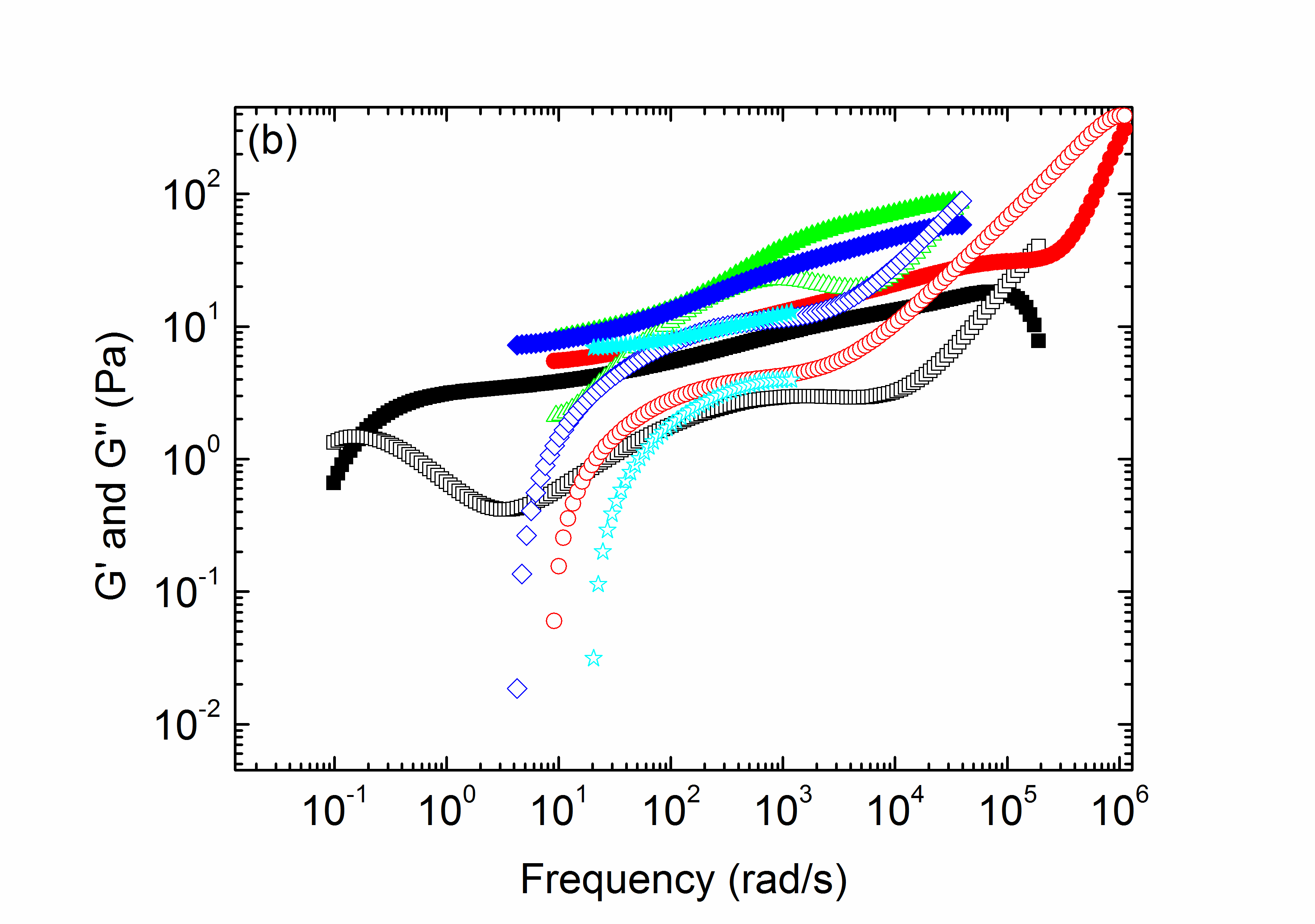


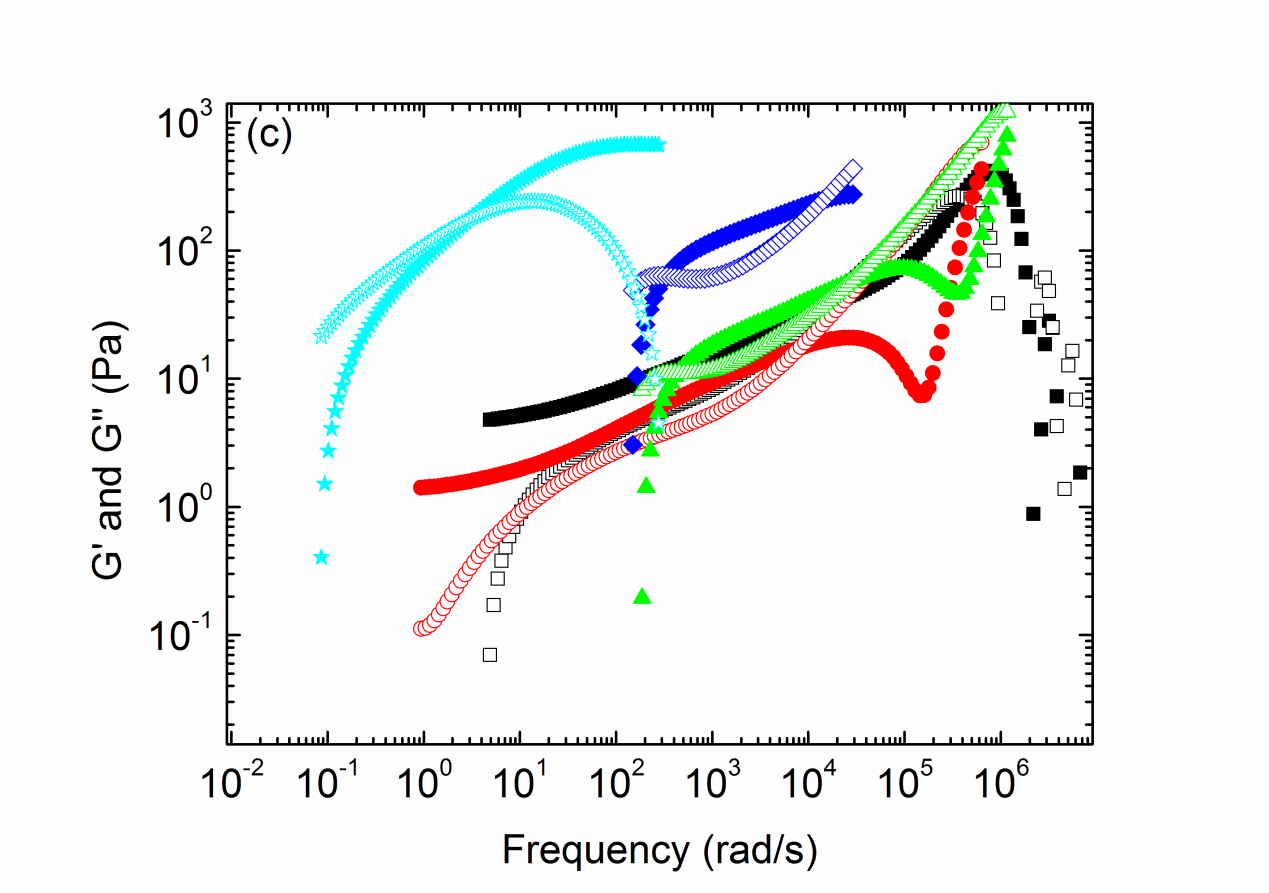


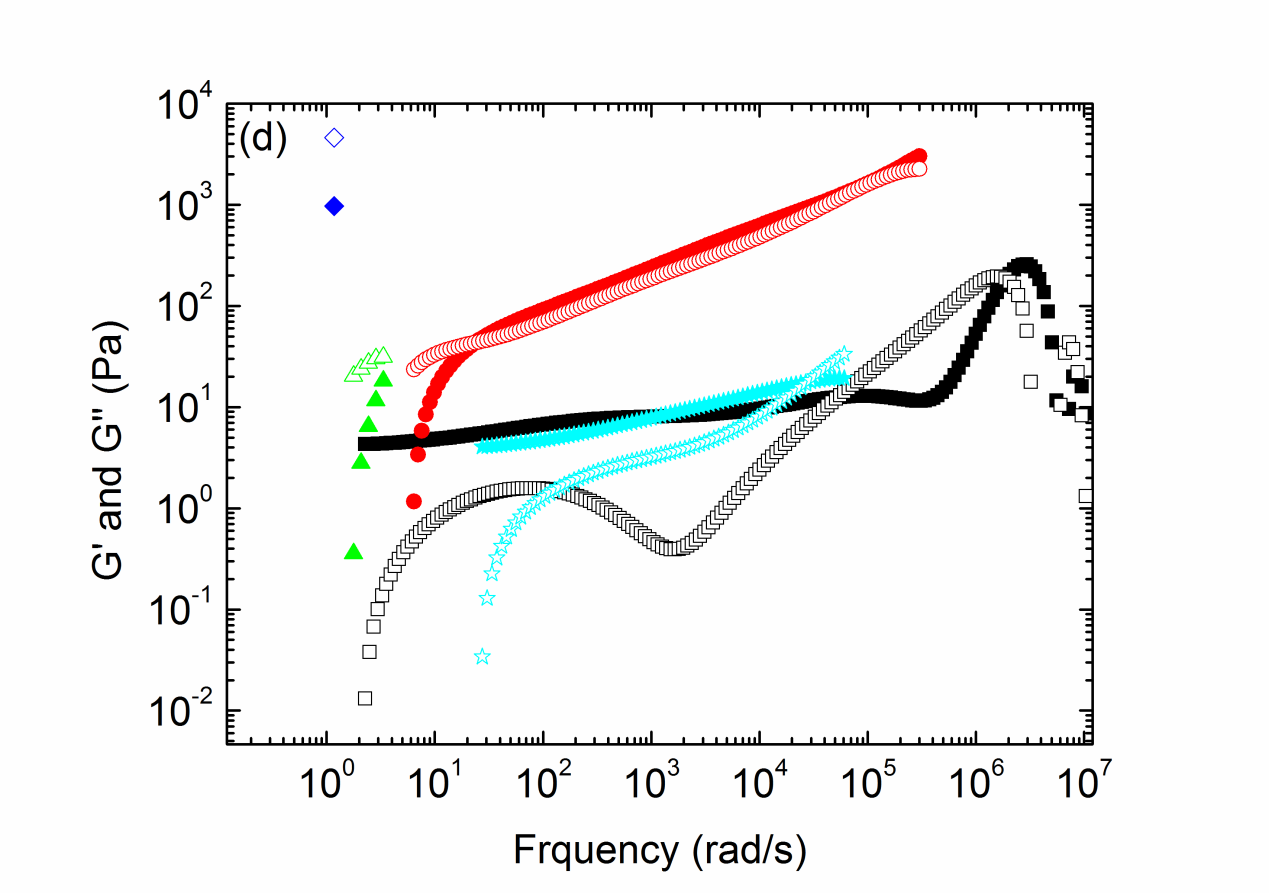


Figure. S7 The elastic modulus (denoted by solid symbol) and viscous ones (done by empty symbol) under 5 temperatures ( 25^o^C denoted by black square, 30^o^C by red circle, 35^o^C by green triangle, 40^o^C by blue diamonds, and 45^o^C by cyan star) at 4 concentrations of KCl (0 mol/L (a), 0.15 mol/L (b), 0.5 mol/L (c), and 1 mol/L (d)).
